# The Preparedness for Caregiving Scale in African American and White Caregivers of Hospitalized Persons With Dementia

**DOI:** 10.1093/geroni/igab046.1449

**Published:** 2021-12-17

**Authors:** Ashley Kuzmik

**Affiliations:** Pennsylvania State University, University Park, Pennsylvania, United States

## Abstract

This study evaluated the Preparedness for Caregiving Scale (PCS) upon discharge from the hospital. The caregivers reported a mean age of 60.5 years (SD=13.9). The majority of caregivers were female (72%), married (59%), non-Hispanic/Latino (98%) and either white (52%) or African American (48%). Fifty percent were employed outside of the home and averaged 40.7 (SD= 14.4) hours of outside work per week. The average PCS was 24.4 (SD=6.9, 0-32). One-factor structure of the PCS and measurement invariance by race was fully supported. Predicative validity revealed significant association between the PCS and anxiety (β =-.41, t = -7.61(287), p <.001), depression (β =-.44, t =-8.39 (287), p <.001), and strain (β =-.48, t =-9.29(287), p <.001). The PCS is a valid and meaningful tool to measure preparedness in African American and white family caregivers of persons with dementia during post- hospitalization transition.

